# Chest x-ray imaging score is associated with severity of COVID-19 pneumonia: the MBrixia score

**DOI:** 10.1038/s41598-022-25397-7

**Published:** 2022-12-05

**Authors:** Christian M. Jensen, Junia C. Costa, Jens C. Nørgaard, Adrian G. Zucco, Bastian Neesgaard, Carsten U. Niemann, Sisse R. Ostrowski, Joanne Reekie, Birgit Holten, Anna Kalhauge, Michael A. Matthay, Jens D. Lundgren, Marie Helleberg, Kasper S. Moestrup

**Affiliations:** 1 Centre of Excellence for Health, Immunity and Infections (CHIP), Section 2100, , Rigshospitalet, University of Copenhagen, Blegdamsvej 9, 2100 Copenhagen Ø, Denmark; 2 Department of Diagnostic Radiology, Rigshospitalet, University of Copenhagen, Copenhagen, Denmark; 3 Department of Haematology, Rigshospitalet, University of Copenhagen, Copenhagen, Denmark; 4 Department of Clinical Medicine, University of Copenhagen, Copenhagen, Denmark; 5 Department of Clinical Immunology, Rigshospitalet, University of Copenhagen, Copenhagen, Denmark; 6 Departments of Medicine and Anaesthesia, Cardiovascular Research Institute, University of California, San Francisco, CA USA; 7 Department of Infectious Diseases, Rigshospitalet, University of Copenhagen, Copenhagen, Denmark

**Keywords:** Viral infection, Respiratory signs and symptoms

## Abstract

Spatial resolution in existing chest x-ray (CXR)-based scoring systems for coronavirus disease 2019 (COVID-19) pneumonia is low, and should be increased for better representation of anatomy, and severity of lung involvement. An existing CXR-based system, the Brixia score, was modified to increase the spatial resolution, creating the MBrixia score. The MBrixia score is the sum, of a rule-based quantification of CXR severity on a scale of 0 to 3 in 12 anatomical zones in the lungs. The MBrixia score was applied to CXR images from COVID-19 patients at a single tertiary hospital in the period May 4th–June 5th, 2020. The relationship between MBrixia score, and level of respiratory support at the time of performed CXR imaging was investigated. 37 hospitalized COVID-19 patients with 290 CXRs were identified, 22 (59.5%) were admitted to the intensive care unit and 10 (27%) died during follow-up. In a Poisson regression using all 290 MBrixia scored CXRs, a higher MBrixia score was associated with a higher level of respiratory support at the time of performed CXR. The MBrixia score could potentially be valuable as a quantitative surrogate measurement of COVID-19 pneumonia severity, and future studies should investigate the score’s validity and capabilities of predicting clinical outcomes.

## Introduction

Multiple clinical presentations have been described in people infected with coronavirus disease 2019 (COVID-19), ranging from asymptomatic infection to severe pneumonia, acute respiratory distress syndrome (ARDS), and severe inflammatory response syndrome^1^, with COVID-19 ARDS being a common cause of death in severe cases, albeit presenting a pattern deviating from typical ARDS criteria^2,3^. Common radiological findings in COVID-19-related pneumonia include bilateral peripheral, mid-basal predominant ground-glass opacities (GGO), or hazy opacities when using computed tomography (CT) or chest x-ray (CXR) imaging, respectively, with progression of consolidations over time^4,5^.

Several chest imaging modalities have shown to be excellent tools for both assessment of disease stage and progression in COVID-19 pneumonia. While CT’s high sensitivity for detection of COVID-19 related lung lesions, especially at disease onset, exceeds that of CXR imaging, a high proportion of these lesions can also be seen using CXR imaging, a modality which is inexpensive, readily available, and offers a portable alternative that can be utilized without the need for in-hospital transportation of potentially infectious patients^6,7^. Consequently, some research groups have developed CXR based scoring systems to be used for quantification of lung lesions related to COVID-19 pneumonia. CXR severity score at the time of admission has shown to be an independent predictor of intubation^8^, while another study by Borghesi et al. found that the maximum score during admission was an independent predictor of in-patient mortality, along with age and immunosuppressive conditions, using their recently developed CXR imaging score, the *Brixia* score^9^.

In this study the primary objective was to develop a modified version of the Brixia score by redefining the score’s severity criteria, as well as increasing the score’s resolution, allowing clinicians and researchers to more clearly assess lung lesions in COVID-19 pneumonia. To evaluate the clinical relevance of the modified Brixia (MBrixia) score, the relationship between the MBrixia score and level of respiratory support was assessed, as a surrogate marker for disease severity, as well as 15 well-established biomarkers of inflammation and organ injury.

## Methods and materials

### Patient and chest x-ray image selection

Data was collected retrospectively on patients hospitalized with COVID-19 at Rigshospitalet, Copenhagen, Denmark. Rigshospitalet is a tertiary referral hospital, and one of only two hospitals in Denmark that treat patients with extracorporeal membrane oxygenation (ECMO). Patients hospitalized at either the department of infectious diseases or the intensive care unit (ICU) at Rigshospitalet during the time period May 4th–June 5th, 2020, with a diagnosis of COVID-19, confirmed by reverse transcriptase polymerase chain reaction, and at least one available CXR image during hospitalization, were eligible for inclusion. All CXR images available for eligible patients, performed at any department or hospital in the Capital Region of Denmark or Region Zealand, after the day of symptom onset up until June 29th, 2020, were scored for the purpose of this study. On days with more than one CXR available for a given patient, only the score of the first CXR image was included in the analyses. The mortality follow-up period was defined as three months after day of admission.

### Modification of the Brixia score

**Figure 1:**
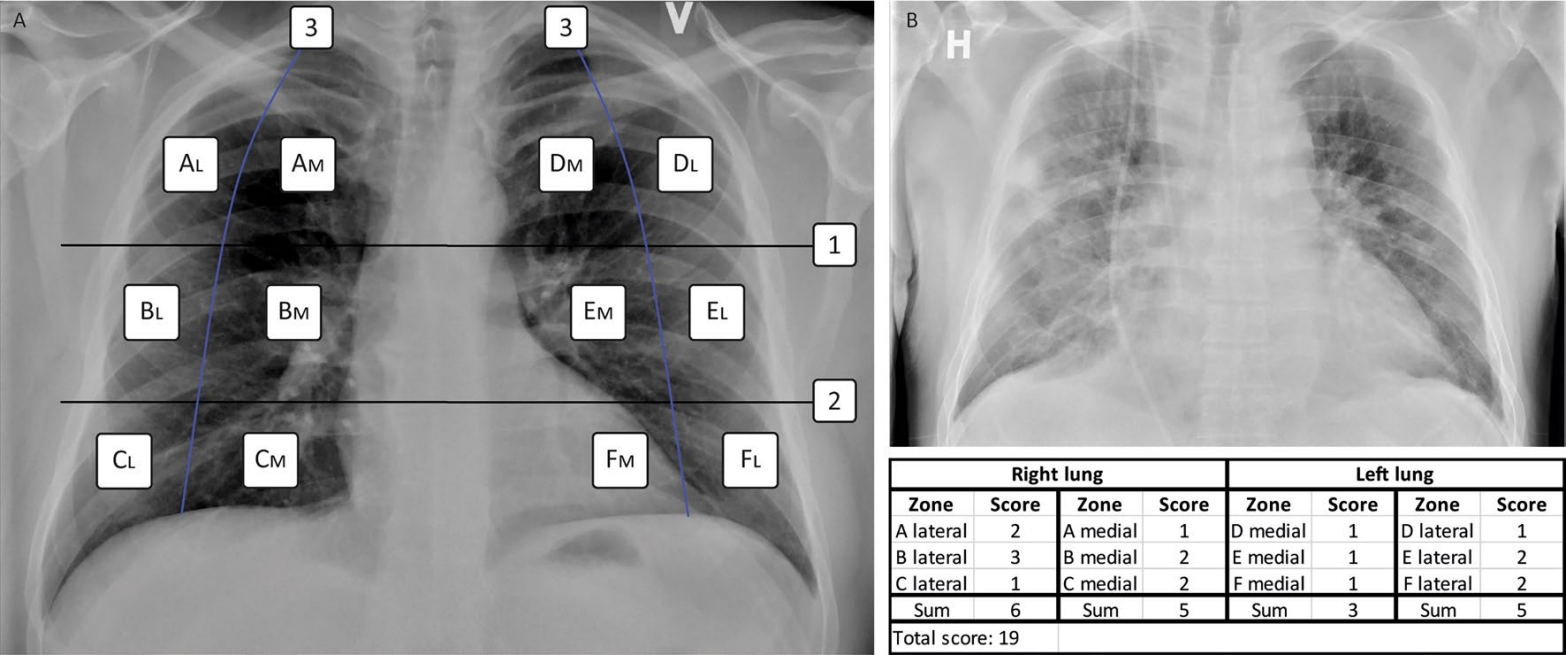
(**A**) The MBrixia scoring system. Box 1 and 2 mark the horizontal division corresponding to the inferior wall of the aortic wall, and the inferior wall of the right pulmonary vein, respectively, creating three zones in each lung: A, B, C, D, E and F. Box 3 marks the vertical line drawn from the pulmonary apices to the diaphragm, creating further division into medial (M) and lateral (L) zones. (**B**) A clinical example of a chest x-ray image scored using the MBrixia score.

As defined in the Brixia score^10^, CXR images in the posteroanterior or anteroposterior projection had each lung divided into three zones, by two horizontal lines: one inferior to the aortic arch and one inferior to the hilar structures. Each zone was then further divided into additional medial and lateral subzones: i.e. medial A, and lateral A; medial B, and lateral B; medial C, and lateral C; medial D, and lateral D; medial E, and lateral E; medial F, and lateral F, by drawing vertical lines from the pulmonary apices to the middle of the diaphragm on each lung. Each vertical line was curved to match the curvature of the ribcage. The resulting 12 individual zones, six in each lung, were scored according to the severity of the lung lesions using a score from zero to three per zone. (Fig. 1).

A score of zero was given if no lung lesions were present in the respective zone, a score of one if interstitial infiltrates were present, a score of two if both interstitial and alveolar infiltrates were present, and a score of three if consolidations were present. In order to divide the findings more clearly, the definition of a score of three was altered from interstitial and alveolar infiltrates with alveolar predominance, as defined in the Brixia scoring system, to consolidations. Each zone was scored according to the most dominant pattern in the respective zone. CXR images of patients suffering from subcutaneous emphysema were assigned the same score as the CXR image scored prior to CXR-verified subcutaneous emphysema. Scores from all 12 zones were summarized into a final score ranging from zero to 36.

The MBrixia score was quickly incorporated into the daily routine at the hospital, and different radiologists independently scored different images. However, the majority of CXR images were scored by three specialized, senior, radiologists, who discussed several cases in order to agree on the systematic approach used in the scoring system.

The rationale of modification was based upon a clinical demand during the onset of the first wave of the COVID-19 pandemic in Denmark. As cases of COVID-19 pneumonia often present with peripheral infiltrates on CXR imaging^5^, the radiologists found the original Brixia score’s definition excessively time-consuming to use in practice, when having to discuss which pattern was dominant in patients with different radiological findings medially versus laterally. The MBrixia score thus allowed the radiologists to reduce and optimize the time spent on scoring CXR images, as well as making it a more reproducible process. Additionally, a further division of each lung meant a theoretically reduced risk of both over- and underestimating the initial assessment as well as the trajectory of the pneumonia, as lower- or higher-scoring dominating patterns in the opposing side of the ipsilateral lung would not be the determinant factor of a zone’s score. This was of particular importance in COVID-19 pneumonia, as certain dominant patterns have been found not to be linked to disease severity^11^. Two clinical examples comparing the Brixia and MBrixia scores can be found in the supplementary files. (Supplementary Fig. 1).

### Data sources

All patient data were extracted from electronic healthcare records and the PERSIMUNE clinical database^12^. Biochemical data included 15 biochemical parameters related to inflammation and organ injury derived from routine blood samples performed during the COVID-19 related hospitalizations: alanine aminotransferase (ALT), creatinine, c-reactive protein (CRP), fibrin d-dimer, fibrinogen, hemoglobin, lactate dehydrogenase (LDH), plasma albumin, plasma ferritin, procalcitonin (PCT), in addition to eosinophil-, lymphocyte-, monocyte-, neutrophil-, and thrombocyte count. The study was approved by the Regional Ethical Committee of the Capital Region of Denmark (H-20026502), and the Data Protection Agency (P-2020-426), and requirement for informed consent was waived for this study. The study was conducted in accordance with the Declaration of Helsinki.

Current and historical International Classification of Diseases, 10th Revision codes were used to identify comorbidities as classified by the Charlson Comorbidity Index (CCI)^13^. The individual codes corresponding to each component of the CCI can be found in the supplementary files. (Supplementary Table 1) Due to the small sample size, diabetes was defined as both diabetes with, and without, end-organ damage, liver disease was defined as both mild, and moderate-severe liver disease, and malignancy was defined as both malignant solid tumors, and hematological malignancies. Components of the CCI with a cumulative frequency of zero were omitted from Table 1.

**Table 1:** Baseline characteristics.

Patients included, n (%)	37 (100.0)
Male sex, n (%)	25 (67.6)
Age, years, median (IQR)	58 (54–65)
BMI, median (IQR)^a^	25.3 (22.8–29.0)
**Comorbidities, n (%)**	
None	16 (43.2)
One or more	21 (56.8)
Malignancy	13 (35.1)
Chronic pulmonary disease	< 5^b^
Chronic renal disease	< 5^b^
Chronic heart failure	< 5^b^
Diabetes mellitus	< 5^b^
Rheumatological disease	< 5^b^
Acute myocardial infarction	< 5^b^
Peripheral vascular disease	< 5^b^
Cerebrovascular disease	< 5^b^

Baseline characteristics of patients hospitalized at the department of infectious diseases or the intensive care unit at Rigshospitalet in the time period May 4th, 2020–June 5th, 2020.

^a^Data on BMI was missing for two patients.

^b^Exact number of patients censored to maintain patient confidentiality due to small number of events.

### Statistical analysis

The pulmonary lesion distribution was assessed using the CXR images performed closest to the time of admission, and in another separate analysis using CXR images at the time of maximum MBrixia score. In both analyses the mean MBrixia score per anatomical zone was calculated. In the case of more than one CXR with identical maximum scores, the distributional pattern of the earliest registered CXR image in the analysis was used. For patients with only one image available, the same image was used in both analyses. CXR images with a score of zero were excluded, as no distribution could be reported.

Respiratory support was categorized according to severity based on an ordinal scale of five levels: no supplemental oxygen; one to five liters of oxygen per minute; more than five liters of oxygen per minute, high-flow nasal cannula (HFNC), or non-invasive ventilation (NIV); invasive mechanical ventilation; and ECMO. Scored CXR images were matched to the level of respiratory support at the time the imaging was performed, by comparing time stamps to electronic healthcare records.

Biochemical values for laboratory tests were summarized in a daily average. CXR images were paired with biochemical values in a prioritized order; scored CXR images were paired with biochemical values of the same date if available. If no same-date pairing was available, CXR images were paired with values from the day before, the day after, or an average of both. Any remaining unmatched scored CXR images were omitted from the respective correlation analysis.

#### Statistical tests

Correlation analyses of MBrixia scores, and the 15 biochemical variables, were assessed using Kendall rank correlation. To reduce the risk of type-1 errors in the main analysis, a p-value < 0.0033 was deemed statistically significant in accordance with the Bonferroni correction method. Since patients had a varying number of MBrixia scored CXR images per patient, sensitivity analyses for each correlation test were tested where each patient had only two randomly chosen MBrixia scored CXR images to check the robustness of the results.

Association between the MBrixia score and the level of respiratory support, and a predicted mean MBrixia score for each level of respiratory support were tested by Poisson regression using generalized estimating equations with robust standard errors to account for potential bias caused by patients having multiple CXR images performed. In a sensitivity analysis, patients that were not eligible for invasive mechanical ventilation or ECMO treatment were excluded. Incidence rate ratios and 95% confidence intervals (CI) were calculated using the least severe level of respiratory support, “no supplemental oxygen”, as reference group.

All statistical analyses were performed using STATA 16.1 (StataCorp, College Station, Texas, USA), SAS Studio Version 9.4 (SAS Institute Inc., Cary, NC, USA) and R 3.6.1 (R Core Team).

### Ethics approval

The study was approved by the Regional Ethical Committee of the Capital Region of Denmark (H-20026502) and the Data Protection Agency (P-2020-426) and requirement for informed consent was waived for this study.

## Results

### Patient characteristics and MBrixia score distribution

A total of 37 patients were included in the study and contributed 290 unique MBrixia scored CXR images (Supplementary Fig. 2). The median number of scored CXR images per patient was 7 (interquartile range [IQR], 3–11) (Supplementary Fig. 3). Two patients had a total of eight CXR images scored while experiencing subcutaneous emphysema.

Baseline characteristics are presented in Table 1. The majority of patients were male (25, 67.6%), the median age was 58 years (IQR 54–65), and 21 (56.8%) had at least one comorbidity. Malignancy was the comorbidity of highest prevalence with 13 (35.1%) having one, or more, malignant diagnoses. Of the 37 patients, 22 (59.5%) were admitted to the ICU, and 10 (27%) died within the follow-up period. The median duration of admission was 44 days (range 1–122 days).

**Figure 2:**
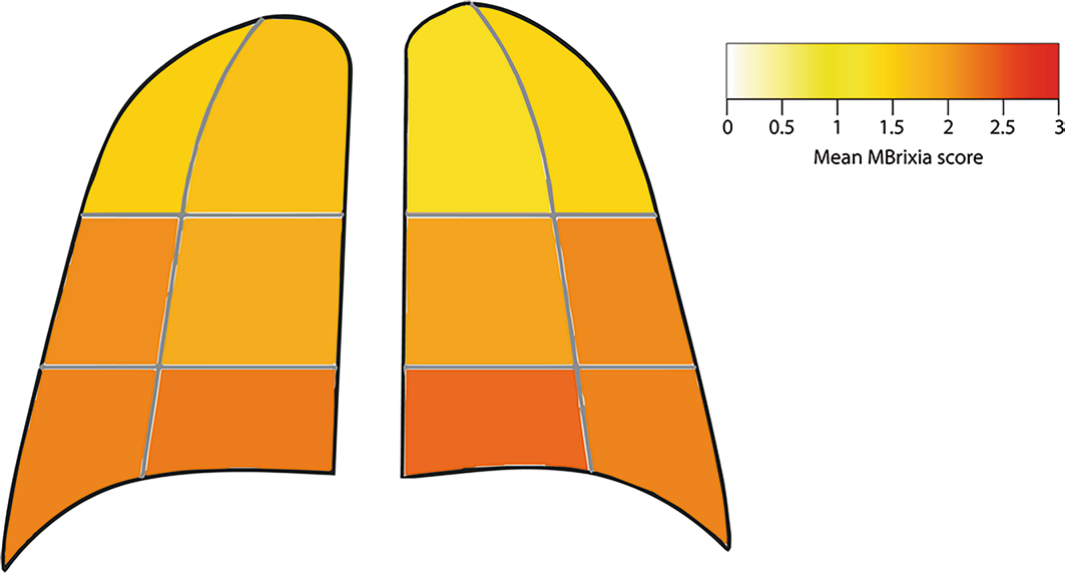
Distribution of lung lesions at time of maximum MBrixia score for 36 patients admitted to the hospital with COVID-19. One patient was left out of the analysis due to having a maximum score of zero.

The distribution of lung lesions was first asssessed using the MBrixia score at the time of admission, and at the time of maximum MBrixia score. The quantified distribution of lung lesions across all 12 pulmonary zones showed bilateral lung tissue involvement with a trend toward mid-basal peripheral predominance. At the time of maximum MBrixia score, the mean MBrixia score of the right, and left lung was 11.58 (95% CI 10.00–13.16) and 11.44 (95% CI 10.02–12.87), respectively, with a mean total MBrixia score of 23.03 (95% CI 20.13–25.92). (Fig. 2) The complete quantification of lung lesions can be found in the supplementary files (Supplementary table 2).

### Correlations between MBrixia score and biomarkers of inflammation and organ injury

To assess potential correlation between inflammatory markers related to COVID-19 infection, and pathological radiological findings, the relationship between MBrixia score, and 15 biomarkers of inflammation and organ injury matched to the time of the performed CXR images was investigated. Positive correlations were found between MBrixia score, and seven of the investigated biomarkers (p < 0.0033): fibrin d-dimer (tau-b = 0.343), neutrophil count (tau-b = 0.311), PCT (tau-b = 0.195), monocyte count (tau-b = 0.192), LDH (tau-b = 0.177), eosinophil count (tau-b = 0.168), and CRP (tau-b = 0.148). There was no correlation with the remaining eight investigated biomarkers. (Table 2 and Supplementary Fig. 4).

**Table 2:** Correlation analyses of MBrixia scored chest x-ray images and 15 time-matched biochemical values.

Biochemical value	Observations (n)	tau-b	p-value
Alanine aminotransferase	236	− 0.001	0.976
Creatinine	249	0.105	0.016
C-reactive protein	250	0.148	< 0.001*
Eosinophil count	163	0.168	0.003*
Fibrin d-dimer	191	0.343	< 0.001*
Fibrinogen	116	− 0.089	0.174
Haemoglobin	247	− 0.091	0.039
Lactate dehydrogenase	235	0.177	< 0.001*
Lymphocyte count	163	0.054	0.332
Monocyte count	163	0.192	< 0.001*
Neutrophil count	147	0.311	< 0.001*
Plasma albumin	220	− 0.035	0.458
Plasma ferritin	186	− 0.006	0.901
Procalcitonin	184	0.195	< 0.001*
Thrombocyte count	250	− 0.016	0.721

*Statistically significant (p-value < 0.0033).

The sensitivity analysis, where data were restricted to two randomly selected scored CXR images per patient, was broadly consistent with the main analysis. However, stronger correlations were observed with LDH (tau-b = 0.177 versus 0.387), plasma albumin (tau-b = − 0.035 versus − 0.188), and ALT (tau-b = − 0.001 versus 0.149). A weaker correlation was observed with monocyte count (tau-b = 0.192 versus 0.083). (Supplementary Table 3).

### The association between MBrixia score, and level of respiratory support

**Figure 3:**
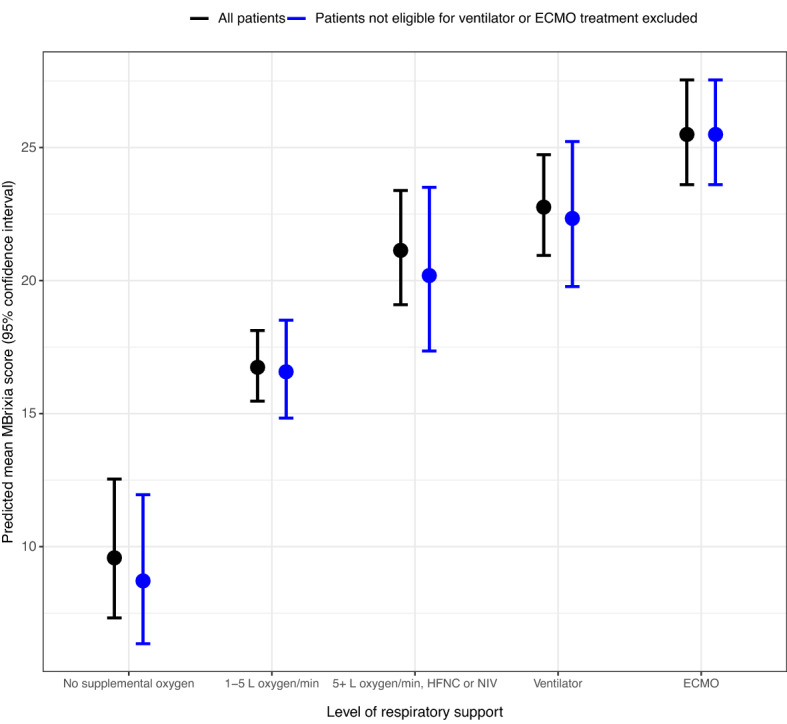
Predicted mean MBrixia score (95% confidence interval) by level of respiratory support.

Finally, the relationship between the MBrixia score, and respiratory support at time of the performed CXR imaging was assessed. A higher MBrixia score was associated with a higher level of respiratory support at the time of CXR in the total cohort, and in a subgroup analysis excluding patients not eligible for invasive mechanical ventilation or ECMO treatment. The median MBrixia score at each level of respiratory support was 8 (IQR 5–15), 18 (15–19), 21 (19–24), 23 (20–26), and 25 (23–27) in patients receiving no supplemental oxygen; one to five liters of oxygen per minute; more than five liters of oxygen per minute, HFNC, or NIV; invasive mechanical ventilation; and ECMO, respectively. (Supplementary Figs. 5, 6) In the Poisson regression model taking multiple observations per patient into account, these differences were found to be significantly different. The mean MBrixia score was estimated to be 1.75 (95% CI 1.31–2.33, p = 0.0002), 2.21 (1.65–2.95, p < 0.0001), 2.38 (1.80–3.14, p < 0.0001), and 2.66 (2.01–3.52, p < 0.0001) times higher in patients receiving one to five liters of oxygen per minute; more than five liters of oxygen per minute, HFNC, or NIV; invasive mechanical ventilation; and ECMO, respectively, when using patients receiving no supplemental oxygen as a reference group. The mean predicted MBrixia score in each category was 9.58 (95% CI 7.32–12.54), 16.74 (15.47–18.12), 21.13 (19.09–23.38), 22.76 (20.94–24.73), and 25.49 (23.60–27.54) in patients receiving no supplemental oxygen; one to five liters of oxygen per minute; more than five liters of oxygen per minute, HFNC, or NIV; invasive mechanical ventilation; and ECMO, respectively. Similar results were found in the subgroup analysis excluding patients not eligible for invasive mechanical ventilation, or ECMO treatment. (Fig. 3) Results for both groups are presented in Table 3.

**Table 3:** Rate ratios and predicted MBrixia scores.

Respiratory support level	All patients	Excluding patients not eligible for mechanical ventilation or ECMO treatment
	Rate ratio [95% CI]	Rate ratio [95% CI]
No supplemental oxygen	1 (Reference)	1 (Reference)
1–5 L of oxygen/min	1.75 [1.31–2.33]	1.90 [1.35–2.69]
> 5 L of oxygen/min, high flow nasal cannula or non-invasive ventilation	2.21 [1.65–2.95]	2.32 [1.64–3.28]
Invasive mechanical ventilation	2.38 [1.80–3.14]	2.56 [1.86–3.54]
Extracorporeal membrane oxygenation	2.66 [2.01–3.52]	2.93 [2.11–4.05]

	Predicted mean score [95% CI]	Predicted mean score [95% CI]
No supplemental oxygen	9.58 [7.32–12.54]	8.71 [6.35–11.95]
1–5 L of oxygen/min	16.74 [15.47–18.12]	16.57 [14.83–18.51]
> 5 L of oxygen/min, high flow nasal cannula or non-invasive ventilation	21.13 [19.09–23.38]	20.19[17.35–23.50]
Invasive mechanical ventilation	22.76 [20.94–24.73]	22.33 [19.77–25.22]
Extracorporeal membrane oxygenation	25.49 [23.60–27.54]	25.49 [23.60–27.54]

## Discussion

In this study of a modified Brixia score in 37 patients with 290 scored CXR images, MBrixia was significantly associated with the level of respiratory support at the time of CXR imaging. Moreover, there was a significantly higher mean MBrixia score at each level of respiratory support when comparing with the scored CXR images of patients receiving no supplemental oxygen, with a noticeable trend of a higher mean MBrixia score at each subsequent level of respiratory support. While previous studies have found an association between quantified pulmonary radiological findings and various clinical outcomes in COVID-19, pneumonia of other viral etiologies, and ARDS-associated lung oedema^9,14–17^, this study demonstrates an association between a CXR-based score for COVID-19 and the level of respiratory support. Interestingly, one study points towards ARDS-transition in COVID-19 already at time of HFNC-requirement, thus raising the question of using MBrixia score as a complementary tool for diagnosing and monitoring ARDS, as chest radiography is already an integrated component of the ARDS Berlin-definition^18,19^.

This study found positive correlations between the MBrixia score and fibrin d-dimer, neutrophil count, PCT, monocyte count, LDH, CRP, and eosinophil count. These biomarkers have all been reported to be of significance in COVID-19 at various stages of disease^20–22^. Fibrin d-dimer, a biomarker of fibrinolytic activity, correlated strongest of all the investigated biomarkers with the MBrixia score. This is in line with the pro-thrombotic phenotype reported in patients with severe COVID-19 disease^23,24^. Additionally, the MBrixia score correlated positively with CRP, PCT, and neutrophil count, all biomarkers associated with poor clinical outcomes, severe disease, and complications such as bacterial superinfections^20,25–27^. These findings support the notion of using the MBrixia score as a proxy for disease severity.

The MBrixia score’s modified definition of both anatomy, and pathology, and its broader scoring range compared to the Brixia score (0–36 points versus 0–18 points), made it possible to capture a more detailed spatial quantification of COVID-19 pneumonia, as seen in Fig. 2. This is of importance, since COVID-19 pneumonia can present with several radiological findings of varying degree, all correlated with clinical outcome^5,28,29^. A traditional division of lung lesions, according to its anatomical location in one of the five lung lobes, was found to be useful for predicting patient outcomes at the time of presenting to the emergency department^30^. However, the MBrixia score allows for quantification of the overall lesion burden, and it is advantageous in COVID-19 pneumonia, since studies using higher-resolution imaging, such as CT imaging, have reported COVID-19 lesions in lobar segments—as opposed to entire lung lobes^31,32^. Furthermore, when comparing the Brixia score with the MBrixia score, as shown in Supplementary Fig. 1, the findings of this study suggest a reduced risk of both over- and underestimating the severity of lung lesions when using the MBrixia score. These modifications thus allow physicians and researchers to track disease status and illness trajectory more accurately, and with more detail.

Current World Health Organization guidelines on clinical care for severe acute lower respiratory infections, such as COVID-19, are based on supplemental oxygen requirement^33^. A strong association between the MBrixia score, and current level of respiratory support was observed in this study. Thus, following external validation, the MBrixia score could be implemented as a supplementary tool in routine clinical care of COVID-19 patients, in line with these guidelines.

Since written radiological descriptions were done routinely at our center, the MBrixia score was implemented as an add-on to radiological routine work, and score results were implemented for descriptive purposes in a clinical application that was used in day-to-day COVID-19 patient care. To minimize the radiologists’ time used on CXR scoring, the MBrixia score can potentially be derived from automated scoring processes following the latest advances in machine-learning. Automated scoring has been investigated by Signoroni et al.^34^, who trained a Brixia score based model in a weakly supervised learning framework, enabling it to predict Brixia scores using CXR images from patients with COVID-19 pneumonia. However, in the case of the MBrixia score, larger studies or routine implementation are needed to provide enough data required for fully automated scoring processes as well as validation studies.

There are some limitations to this study. A sample size of 290 scores taken from 37 patients with a varying range of scored CXR images per patient could lead to an overrepresentation, and enrichment of correlations, as patients with more severe disease progression had a higher amount of available CXR images, due to the clinical utilization of CXR imaging. However, sensitivity analyses found consistent results when using a maximum of two MBrixia scores per patient in most correlations, indicating that potential overrepresentation of patients with multiple scores is not critical.

Due to our centre being a tertiary referral hospital with multiple specialized functions, such as treating immunosuppressed patients, and being one of only two hospitals in Denmark providing ECMO treatment, the cohort in this study may not represent the general population of patients being admitted due to COVID-19 and related complications. The cohort does, however, represent hospitalizations that lie on the entire spectrum of severity, with some patients being discharged shortly after admission, and others being hospitalized for over a month. Additionally, patients with a current or historical diagnosis of malignancy may be overrepresented in the cohort. This overrepresentation may however be partly explained by the inclusion period, as it corresponds to the end stages of the first wave of COVID-19 in Denmark. Anecdotally, a similar pattern of hospitalized COVID-19 patients with certain comorbidities was seen at our centre during the second wave of COVID-19. Lastly, assessing the intra- and interobserver variability of a radiologically based score, as well as external validation in cohorts of diverse ancestry is needed to validate the score. However, this was not within the scope of this study.

Current literature provides evidence of using characteristic imaging features, such as hazy opacifications, GGO, and consolidations at time of first hospital presentation as predictors of inferior outcomes^9,35,36^. However, the small sample size of this study unfortunately provided insufficient statistical power to assess the MBrixia score’s capability to predict clinical outcomes. Future studies are being planned to investigate this.

## Conclusion

The MBrixia score delivered a high-resolution quantification of COVID-19 pneumonia, was associated with current level of respiratory support in patients hospitalized with COVID-19, and correlated positively with seven biomarkers of inflammation and organ injury, all known to be associated with severe COVID-19. Therefore, the use of MBrixia score in the clinical setting is expected to prove beneficial in tracking progression of COVID-19 pneumonia over time, as well as improving healthcare personnel’s understanding, and communication regarding COVID-19 patient care. Larger studies are needed for validation and assessment of the MBrixia score’s predictive capabilities for mortality and disease progression.

## Supplementary Information

Supplementary Information.

## Data Availability

Data available from the corresponding author upon reasonable request.

## Abbreviations

ALT: Alanine aminotransferase
ARDS: Acute respiratory distress syndrome
CCI: Charlson comorbidity index
CI: Confidence interval
COVID-19: Coronavirus disease 2019
CRP: C-reactive protein
CT: Computed tomography
CXR: Chest x-ray
ECMO: Extracorporeal membrane oxygenation
GGO: Ground-glass opacity
HFNC: High-flow nasal cannula
ICU: Intensive care unit
IQR: Interquartile range
LDH: Lactate dehydrogenase
MBrixia score: Modified Brixia score
NIV: Non-invasive ventilation
PCT: Procalcitonin
