# Supplementary material for: Chest x-ray imaging score is associated with severity of COVID-19 pneumonia: the MBrixia score
Source: Sci Rep. 2022 Dec 5;12:21019. doi: 10.1038/s41598-022-25397-7 (PMC9722655; doi:10.1038/s41598-022-25397-7)
Supplement: Supplementary file 1 — Supplementary Information. [file 41598_2022_25397_MOESM1_ESM.docx]

**Supplementary information**

**Title**: Chest x-ray imaging score is associated with severity of COVID-19 pneumonia: the MBrixia score

Author list: *Christian M. Jensen, BSc^1^; Junia C. Costa, MD^2^; Jens C. Nørgaard, MD^1^; Adrian G. Zucco, MSc^1^; Bastian Neesgaard, MD PhD^1^; Carsten U. Niemann, MD PhD^3,4^; Sisse R. Ostrowski, MD PhD DMSc^4,5^; Joanne Reekie, PhD^1^; Birgit Holten, MD^2^; Anna Kalhauge, MD^2^; Michael A. Matthay, MD^6^; Jens D. Lundgren, MD DMSc^1,4,7^; Marie Helleberg, MD PhD DMSc^1,7^; Kasper S. Moestrup, MD^1^

**Affiliations:**

^1^ Centre of Excellence for Health, Immunity and Infections (CHIP), Section 2100, Rigshospitalet, University of Copenhagen, Copenhagen, Denmark

^2^ Department of Diagnostic Radiology, Rigshospitalet, University of Copenhagen, Copenhagen, Denmark

^3^ Department of Haematology, Rigshospitalet, University of Copenhagen, Copenhagen, Denmark

^4^ Department of Clinical Medicine, University of Copenhagen, Copenhagen, Denmark

^5^ Department of Clinical Immunology, Rigshospitalet, University of Copenhagen, Copenhagen, Denmark

^6^ Departments of Medicine and Anaesthesia, Cardiovascular Research Institute, University of

California, San Francisco, CA, USA

^7^ Department of Infectious Diseases, Rigshospitalet, University of Copenhagen, Copenhagen, Denmark

***Corresponding Author contact information**:

Christian M. Jensen, BSc

Cell: +45 22746079, fax number: +45 36473340

[christian.moeller.jensen@regionh.dk](mailto:christian.moeller.jensen@regionh.dk)

Rigshospitalet, CHIP, Section 2100

Blegdamsvej 9, 2100 Copenhagen Ø, Denmark

**
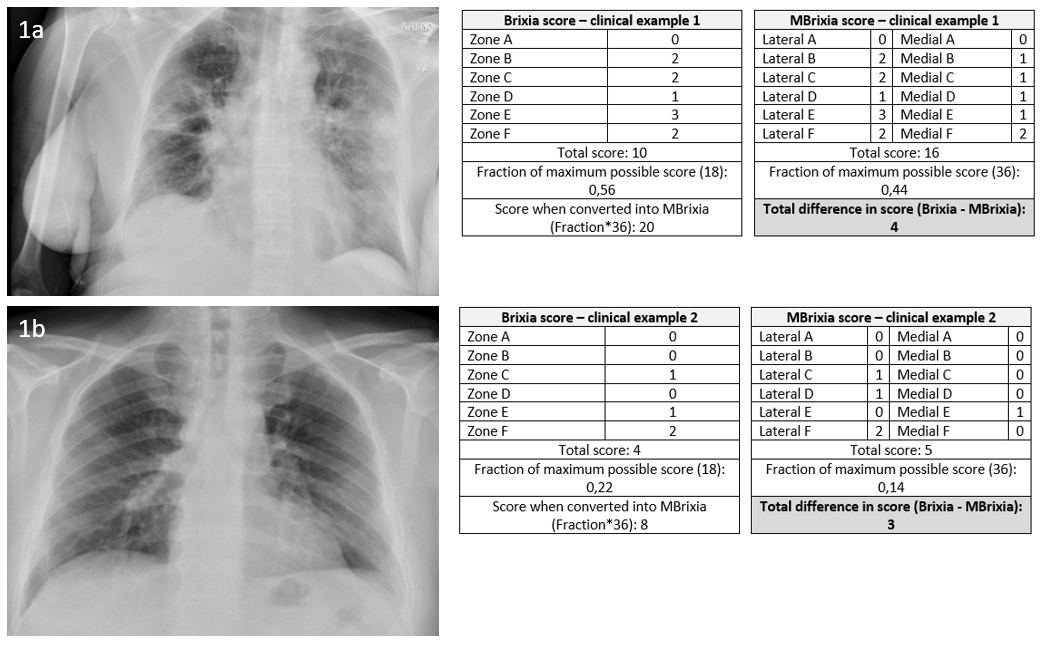
**

**Supplementary figure 1:** Brixia and MBrixia score comparison; Comparison of the Brixia and the MBrixia scores using clinical examples of chest x-ray images from two patients with COVID-19 pneumonia.

| **Supplementary table 1** – Overview of ICD-10 codes used to identify diagnoses for each component of the CCI. | |
| --- | --- |
| **Comorbidity** | **ICD-10 codes** |
| Acute myocardial infarction | I21* I22* I252* |
| Chronic heart failure | I43* I50* I425* I426* I427* I428* I429* I099* I110* I130* I132* I255* I420* P290* |
| Peripheral vascular disease | I70* I71* I731* I738* I739* I771* I790* I792* K551* K558* K559* Z958* Z959* |
| Cerebrovascular disease | G45* G46* I60* I61* I62* I63* I64* I65* I66* I67* I68* I69* H340* |
| Dementia | G30* F00* F01* F02* F03* F051* G311* |
| Chronic pulmonary disease | J40* J41* J42* J43* J44* J45* J46* J47* J60* J61* J62* J63* J64* J65* J66* J67* I278* I279* J684* J701* J703* |
| Rheumatological disease | M05* M06* M32* M33* M34* M315* M351* M353* M360* |
| Peptic ulcer | K25* K26* K27* K28* |
| Liver disease (mild & moderate-severe) | B18* K73* K74* K700* K701* K702* K703* K713* K714* K715* K762* K763* K764* K709* K717* K760* K768* K769* Z944* I850* I859* I864* I982* K704* K711* K721* K729* K765* K766* K767* |
| Diabetes mellitus (with and without end-organ damage) | E100* E101* E106* E108* E109* E110* E111* E116* E118* E119* E120* E121* E126* E128* E129* E130* E131* E136* E138* E139* E140* E141* E146* E148* E149* E102* E103* E104* E105* E112* E113* E114* E115* E122* E123* E124* E125* E132* E133* E134* E135* E142* E143* E144* E145* E107* E117* E127* E137* E147* |
| Hemiplegia | G81* G82* G830* G831* G832* G833* G834* G041* G114* G801* G802* G839* |
| Chronic renal disease | N18* N19* N032* N033* N034* N035* N036* N037* N052* N053* N054* N055* N056* N057* Z490* Z491* Z492* I120* I131* N250* Z940* Z992* |
| Malignancy | C88* C43* C00* C01* C02* C03* C04* C05* C06* C07* C08* C09* C10* C11* C12* C13* C14* C15* C16* C17* C18* C19* C20* C21* C22* C23* C24* C25* C26* C30* C31* C32* C33* C34* C37* C38* C39* C40* C41* C45* C46* C47* C48* C49* C50* C51* C52* C53* C54* C55* C56* C57* C58* C60* C61* C62* C63* C64* C65* C66* C67* C68* C69* C70* C71* C72* C73* C74* C75* C76* C81* C82* C83* C84* C85* C90* C91* C92* C93* C94* C95* C96* C97* |
| Metastatic tumour | C77* C78* C79* C80* |
| Human immunodeficiency virus | B24* B20* B21* B22* |
| Asterisks indicate that every ICD-10 code starting with the code stated prior to it is included, regardless of the following subcode. | |

**Supplementary table 1**: Overview of ICD-10 codes used to identify diagnoses for each component of the Charlson Comorbidity Index.


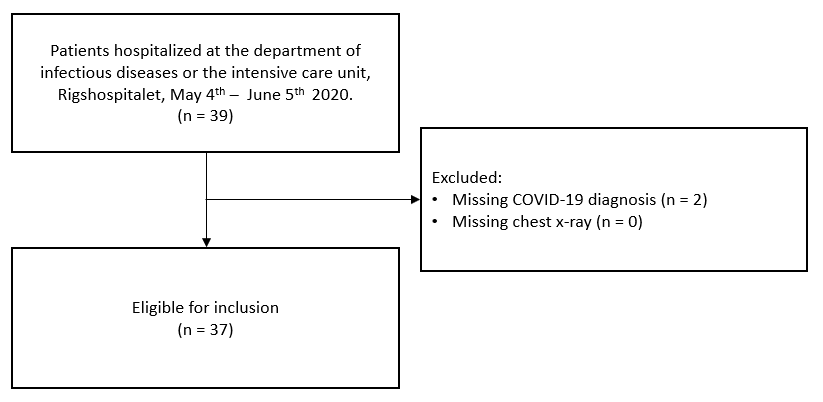


**Supplementary figure 2:** Consort diagram including inclusion and exclusion criteria


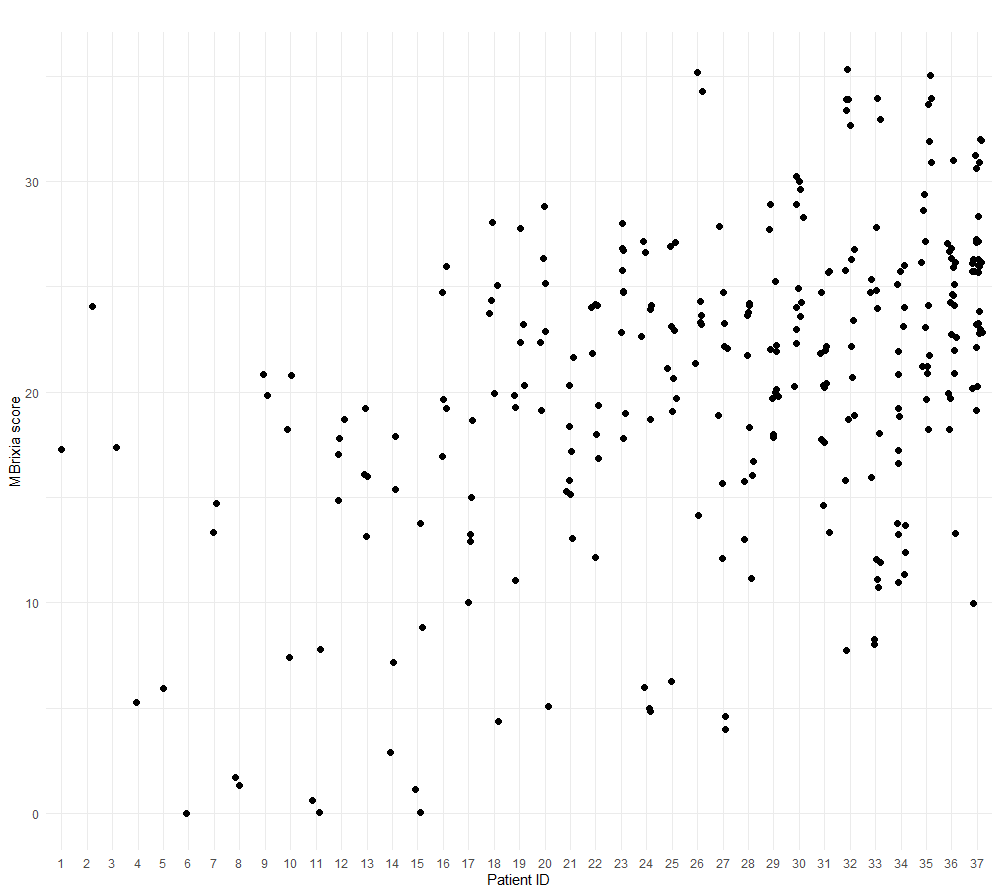


**Supplementary figure 3**: Distribution of MBrixia score (0 to 36 points) per patient. n = 37, observations = 290

| **Supplementary table 2 -** Mean MBrixia score (95% CI) per zone in patients with COVID-19 pneumonia at different time points | | | |
| --- | --- | --- | --- |
| **Using chest x-ray closest to admission (n = 35, two patients omitted from analysis)** | | | |
| **Right lung** | | **Left lung** | |
| Lateral A  Lateral B  Lateral C | 0.80 (0.57 – 1.03)  1.26 (0.98 – 1.54)  1.23 (0.96 – 1.49) | Lateral D  Lateral E  Lateral F | 0.69 (0.44 – 0.93)  1.17 (0.88 – 1.47)  1.46 (0.20 – 1.71) |
| Medial A  Medial B  Medial C | 0.91 (0.66 – 1.17)  1.06 (0.81 – 1.31)  1.26 (0.98 – 1.54) | Medial D  Medial E  Medial F | 0.51 (0.32 – 0.71)  0.97 (0.68 – 1.27)  1.34 (1.09 – 1.59) |
| Right lung mean MBrixia score: 6.52 (5.31 – 7.72) | | Left lung mean MBrixia score: 6.14 (4.94 – 7.35) | |
| Mean total MBrixia score: 12.66 (10.45 – 14.86) | | | |
| **Using chest x-ray at time of max score (n = 36, one patient omitted from analysis)** | | | |
| **Right lung** | | **Left lung** | |
| Lateral A  Lateral B  Lateral C | 1.53 (1.20 – 1.86)  2.11 (1.80 – 2.42)  2.17 (1.88 – 2.45) | Lateral D  Lateral E  Lateral F | 1.47 (1.16 – 1.78)  2.14 (1.83 – 2.44)  2.19 (1.95 – 2.43) |
| Medial A  Medial B  Medial C | 1.67 (1.35 – 1.98)  1.89 (1.58 – 2.20)  2.22 (1.94 – 2.50) | Medial D  Medial E  Medial F | 1.31 (1.04 – 1.57)  1.97 (1.68 – 2.27)  2.36 (2.10 – 2.62) |
| Right lung mean MBrixia score: 11.58 (10.00 – 13.16) | | Left lung mean MBrixia score: 11.44 (10.02 – 12.87) | |
| Mean total MBrixia score: 23.03 (20.13 - 25.92) | | | |
| Patients whose respective chest x-ray had a score of zero were omitted from analyses. | | | |

**Supplementary table 2**: Mean MBrixia score per zone at two different time points


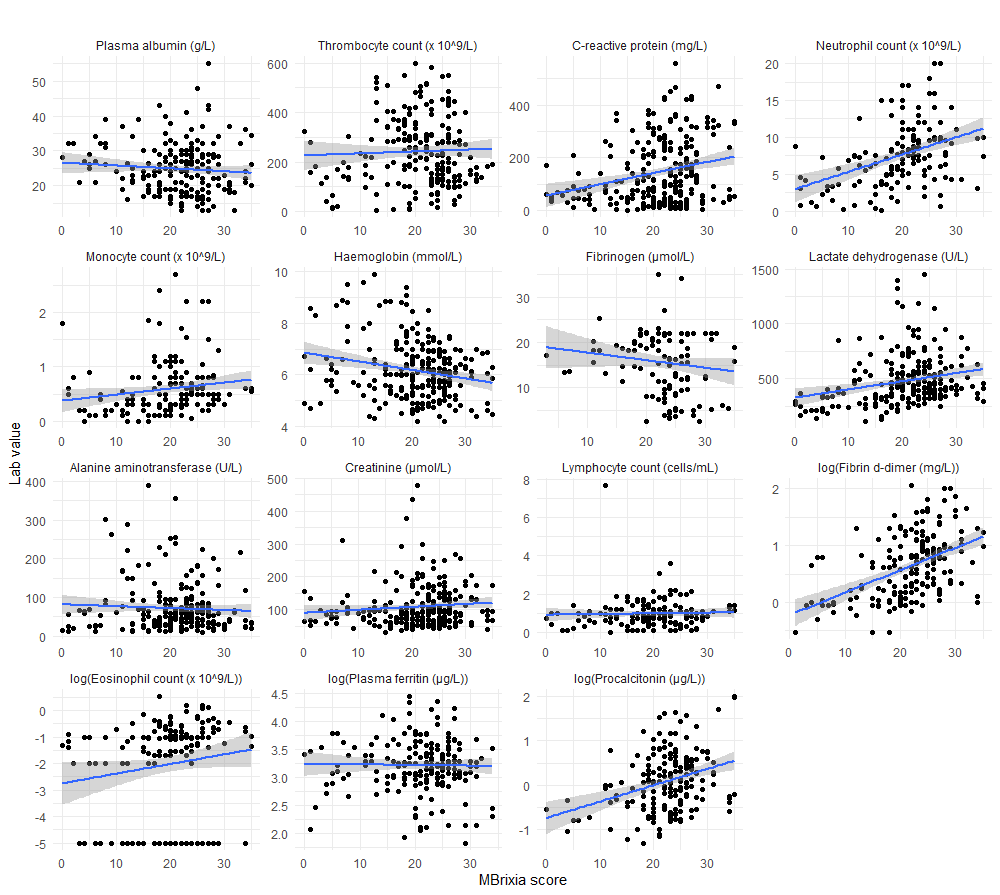


**Supplementary figure 4:** Relationship between MBrixia score (0-36) and 15 biochemical parameters

| **Supplementary table 3 –** Sensitivity analyses of Kendall rank correlations. 2≥ MBrixia scores and matched biochemical value per patient. | | | |
| --- | --- | --- | --- |
| **Biochemical value** | **Observations (n)** | **tau-b** | **p-value** |
| Alanine aminotransferase | 61 | .149 | .097 |
| C-reactive protein | 62 | .227 | .011 |
| Creatinine | 62 | .141 | .112 |
| Eosinophil count | 60 | .123 | .196 |
| Fibrin d-dimer | 51 | .388 | < .001 |
| Fibrinogen | 35 | -.028 | .829 |
| Haemoglobin | 62 | -.155 | .083 |
| Lactate dehydrogenase | 61 | .387 | < .001 |
| Lymphocyte count | 60 | -.033 | .724 |
| Monocyte count | 60 | .083 | .375 |
| Neutrophil count | 56 | .327 | < .001 |
| Plasma albumin | 58 | -.188 | .045 |
| Plasma ferritin | 56 | .040 | .676 |
| Procalcitonin | 45 | .264 | .013 |
| Thrombocyte count | 62 | .064 | .473 |

**Supplementary table 3:** Kendall-Rank correlation analyses for all 15 biochemical variables restricted to a maximum of 2 values per patient


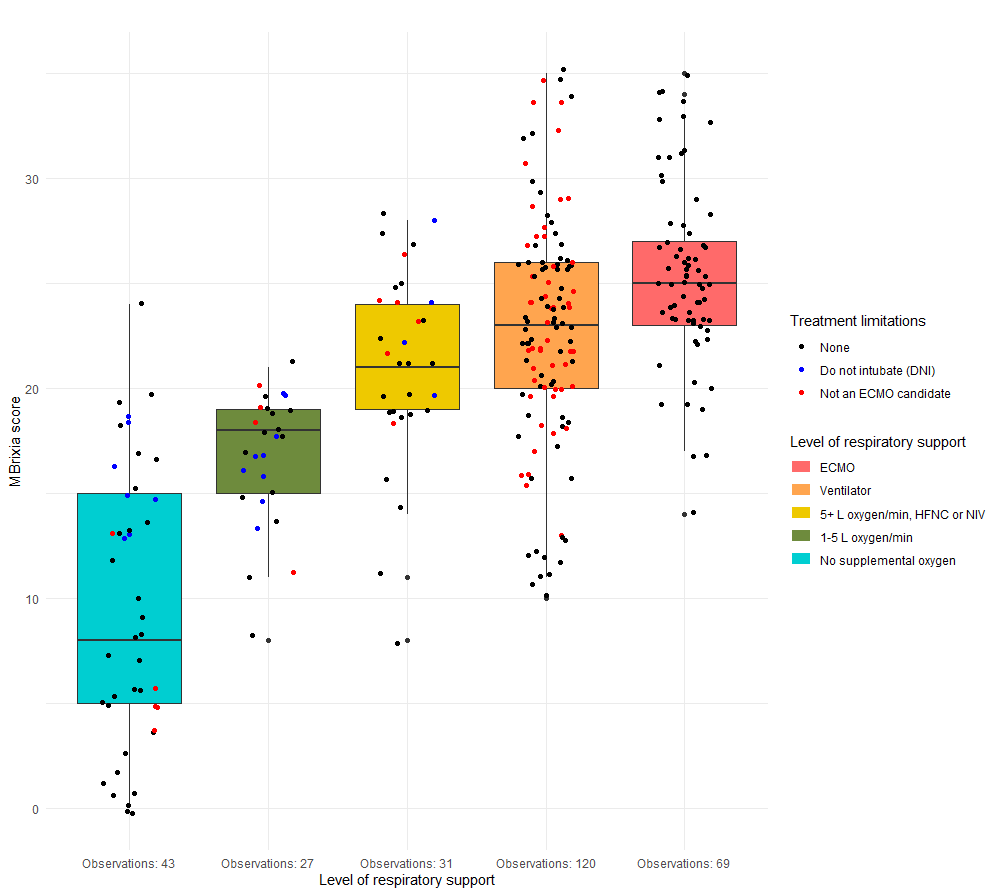


**Supplementary figure 5**: Distribution of MBrixia scores (0 to 36 points) among different levels of respiratory support. n = 37, observations = 290


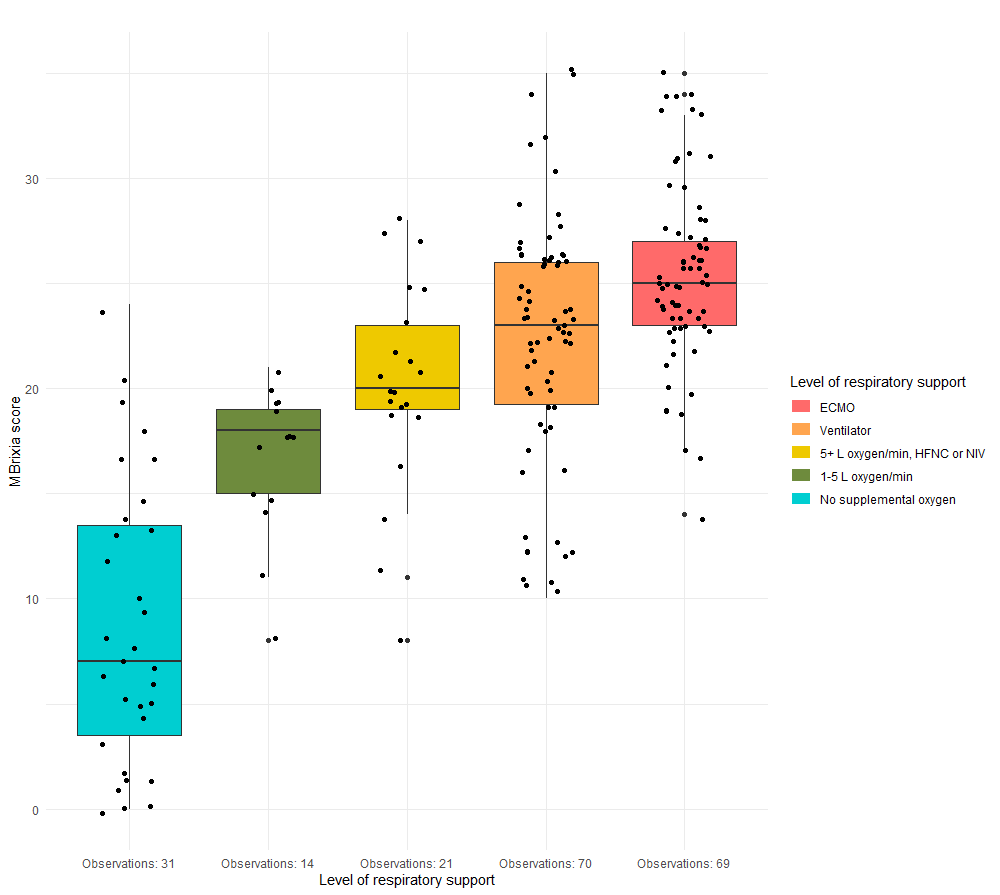


**Supplementary figure 6:** Distribution of MBrixia scores (0 to 36 points) among different levels of respiratory support. Patients ineligible for ventilator or ECMO treatment excluded. n = 26, observations = 205
